# Mesoporous Ag-functionalized magnetic activated carbon-based agro-waste for efficient removal of Pb(II), Cd(II), and microorganisms from wastewater

**DOI:** 10.1007/s11356-023-26000-w

**Published:** 2023-03-02

**Authors:** Omnia I. Ali, Eman R. Zaki, Mohga S. Abdalla, Saber M. Ahmed

**Affiliations:** 1 Chemistry Department, Faculty of Science, Helwan University, Cairo, 11795 Egypt; 2 Soil, Water and Environment Research Institute, Agriculture Research Centre, Giza, Egypt

**Keywords:** Agro-waste, Activated carbon, Magnetite, Ag nanoparticles, Heavy metals, Removal, Antimicrobial activity

## Abstract

Herein, eco-friendly mesoporous magnetic activated carbon-based agro-waste nanosorbents incorporating antimicrobial silver nanoparticles (Mag@AC1-Ag and Mag@AC1-Ag) have been prepared. Various techniques (XRD, SEM/EDX, TEM, FTIR, and BET analysis) were employed to characterize the prepared nanosorbents before being utilized as novel nanosorbents to remove Pb^+2^ and Cd^+2^ ions. Mag@AC1-Ag and Mag@AC1-Ag exhibited rapid and excellent uptake of Pb^+2^ and Cd^+2^. The pseudo-second-order kinetics and the Langmuir isotherm are more suitable for the explanation of the experimental results. The thermodynamic parameters showed that the Pb^+2^ and Cd^+2^ sorption by the nanosorbents was a spontaneous and endothermic reaction. The prepared nanosorbents can be effectively regenerated using HCl and recycled up to the fifth cycle. These nanosorbents’ potential uses for eliminating Pb^+2^ and Cd^+2^ from real water samples were evaluated. Moreover, the results revealed that both Mag@AC1-Ag and Mag@AC2-Ag exhibited high antimicrobial activity against fecal coliform (gram-negative) and *Bacillus subtilis* (gram-positive).

**Supplementary Information:**

The online version contains supplementary material available at 10.1007/s11356-023-26000-w.

## Introduction

Water is a vital necessity for survival, and providing pure water to human beings is one of the prospective development goals to be achieved up to 2030. Rapid industrial and urban growth has contributed to the generation of huge quantities of municipal and industrial wastewater that are contaminated with diverse toxic metals, organic substances, and pathogenic microorganisms. Water pollution caused by industrial, agricultural, and municipal activities has become a serious threat to human and ecosystem environments (Patel 2020; Zhang et al. 2016). Effluents discharged from industrial wastewater contain high concentrations of heavy metals (Thilakan et al. 2022), causing a serious threat to aquatic ecosystems and health problems as they are not biodegradable, toxic in nature, carcinogenic, and can accumulate in the food chain (Li et al. 2019; Wang et al. 2020). Among these metals, cadmium is discharged into the environment as a consequence of cadmium electroplating, phosphate fertilizers, Ni–Cd battery manufacture, cement manufacture, and steel production (Jain et al. 2018). It causes a number of irregularities in humans, including renal degradation, muscular cramps, proteinuria, pulmonary problems, and testicular atrophy (Zaini et al. 2009; Asuquo et al. 2017). Lead is another heavy metal that enters the environment as a result of metal corrosion, fabrications, batteries, gasoline, paints, and pigments (Bahadır et al. 2014). It accumulates in human and animal tissues and causes damage to the brain, kidney, liver, reproductive, and nervous systems and may cause severe health issues, such as abortion, sterility, neonatal deaths, and stillbirths (Parlayıcı and Pehlivan 2017). It interferes with the metabolism of vitamin D and calcium, affects the formation of hemoglobin, and causes anemia (Khajeh 2011). Based on the Environmental Protection Agency, the maximum concentration of cadmium is 0.005 mg L^−1^ and that of lead is 0.015 mg L^−1^ in drinking water (Abatal et al. 2021). Therefore, at even trace concentrations, they have chronic consequences for human beings and plants.

Various technologies have been developed to remove heavy metals from wastewater. Among these technologies, adsorption is quite promising because of its high effectiveness, easy handling, the availability of various sorbent materials, and cost effectiveness (Mia et al. 2017). Activated carbon is a potent adsorbent that is employed to remove dangerous metals because of its large surface area, complex porous structure, high catalytic activity, and the presence of a wide spectrum of functional groups on its surface (Furlan and Melcer 2014). Activated carbon provides a high capacity for adsorption, a high level of surface reactivity, and a powerful affinity for contaminants at even low concentrations. In recent decades, agricultural by-products, including cotton stalk (El Zayat and Smith 2013; Akperov and Akperov 2019), sugarcane bagasse (Mohamed et al. 2015), banana peels (Zhou et al. 2017), rice straw (Zhang et al. 2018), palm shell (Issabayeva et al. 2006; Baby et al. 2019), coconut shell (Chandana et al. 2020), strawberry seeds and pistachio shells (Blachnio et al. 2020), and cotton seed shell (Uçar and Armağan 2012) have been applied as precursors to prepare various activated carbon adsorbents.

Palm shell (also known as endocarp) is a low-cost and widely available agricultural by-product in Egypt. It has been effectively converted into a well-developed activated carbon (Adinata et al. 2007; Yuliusman et al. 2017). Palm shell contains cellulose (26.6%) and hemicelluloses (27.7%), which is suitable to be employed as activated carbon to remove various pollutants (Wong et al. 2016; Baby et al. 2019; Kyi et al. 2020). Although activated carbons show maximum adsorption capacity for heavy metal removal, their use on a large scale is limited because it is notoriously difficult to separate from the solution. Iron oxide nanoparticles are great adsorbents as they have outstanding magnetic characteristics, good reusability, great biocompatibility, and a comparatively low cost (Dave and Chopda 2014; Geneti et al. 2022). A combination of activated carbon and iron oxide nanoparticles (e.g., magnetite and γ-Fe_2_O_3_) has been widely used to synthesize magnetic nanocomposites for easy separation and recovery (Zhang et al. 2018; Fatimah et al. 2021).

Water can be contaminated by numerous organisms, including viruses, bacteria, protozoa, and helminths (Jalali et al. 2017). Waterborne diseases are a global health threat, leading to mortality, and the high cost of their prevention and treatment make it essential to treat wastewater before disposal. Different disinfection techniques can be used to kill pathogens, but unfortunately, the conventional methods, such as chlorination, ultraviolet treatment, and ozonation, have their limitations. For example, chemical disinfectants can react with a number of the natural water constituents, developing disinfection by-products (DBPs) (Mezgebe et al. 2020), many of which are carcinogens (Furlan et al. 2017). The use of antimicrobial nanomaterials can be linked to broad-spectrum activity and is not expected to form harmful DBPs. Among these materials, Ag-based nanoparticles (AgNPs) have strong antimicrobial activity, low toxicity to humans, and no negative effects on the soil community (Najafpoor et al. 2020). As known, the silver nanoparticles’ agglomeration may weaken their ability to disinfect. To control this, silver nanoparticles are incorporated into an adsorbent host matrix, providing high dispersion and preventing the aggregation of incorporated silver nanoparticles. Activated carbon can represent the most hopeful hosting material since it is an effective adsorbent for the removal of a number of contaminants (Furlan et al. 2017). The combination of Ag nanoparticles with magnetic activated carbon permits the antimicrobial materials to be regenerated easily and reused for the preceding cycles of water disinfection, limiting environmental impacts and conserving materials.

In this study, different antimicrobial activated carbon-based agro-waste nanosorbents incorporating magnetite and silver nanoparticles (AgNPs) have been prepared. Activated carbon was prepared from palm shell using two different routes of chemical activation. For the preparation of these bifunctional nanosorbents, magnetite nanoparticles were first immobilized on the prepared activated carbon samples to impart magnetic properties to them. Moreover, to add the biocidal properties to the magnetic activated carbon, AgNPs were integrated onto the previous intermediate products. This study covered the nanosorbents’ preparation, characterization, and batch-mode experiments, which were performed to remove Pb^+2^ and Cd^+2^ from water and wastewater. The kinetics, equilibrium, thermodynamics, and reusability were also explored. Furthermore, the antimicrobial efficacy of the prepared nanosorbents for water disinfection was evaluated.

## Experimental

### Materials

Palm shell was provided by the Faculty of Agriculture, Cairo University, Egypt. KOH, NaOH, and FeSO_4_·7H_2_O were obtained from the Central Drug House (P) Ltd., India. K_2_CO_3_ and FeCl_3_ were purchased from Qualikems Fine Chem. Pvt. Ltd., India. Pb(NO_3_)_2_ and CdCl_2_ were purchased from LOBA Chemie, India. AgNO_3_, fecal coliform, and *Bacillus subtilis* strains were kindly provided by the Soils, Water, and Environmental Res. Inst. (ARC) in Giza, Egypt.

### Preparation of nanosorbent*s*

#### Preparation of activated carbon from palm shell

Palm shell (PS) was washed, dried, and crushed using a steel mill into a fine powder. Six grams of the crushed powder were blended with a KOH or K_2_CO_3_ solution (20 mL, 30%, w/v). The mixture was left overnight in the oven at 120 °C before being transferred to the muffle furnace at 500 °C for 2 h. To remove the residual alkalis, the produced activated carbon (AC) was washed thoroughly with HCl (0.1 mol L^−1^), hot, and cold distilled water until neutral pH. After all, the samples were dried at 105 °C overnight. ACs that were activated chemically with KOH and K_2_CO_3_ were denoted as AC1 and AC2, respectively.

#### Preparation of magnetite/activated carbon nanosorbents and mesoporous magnetite nanoparticles

The mesoporous magnetite/activated carbon nanosorbents were prepared as reported by Jain et al. (2018). One gram of AC was distributed in a 200 mL solution comprising FeCl_3_·6H_2_O (3.9 g) and FeSO_4_·7H_2_O (1.9 g) by vigorous stirring at 60 °C. This solution was dispersed by periodic probe sonication for 10 min. For the precipitation of the hydrated iron oxide, NaOH (10.0 mol L^−1^) solution was added dropwise into the prepared suspension, while vigorous stirring was carried out at 60 °C until the pH reached 10–11. The suspension was then aged for 24 h at room temperature after being stirred for 2 more hours at 60 °C. Then, the formed nanosorbents (Mag@AC1 and Mag@AC2) were separated using a magnet and repeatedly rinsed with distilled water until the pH was neutral. Furthermore, similar to the procedures described above but without the addition of AC, magnetite nanoparticles were synthesized.

#### Synthesis of Mag@AC1-Ag and Mag@AC2-Ag

Practically, the AgNPs synthesis was based on the traditional reduction approach of Lee and Meisel (1982). In brief, 250 mL of AgNO_3_ solution (0.005 mol L^−1^) was heated until it started to boil. Then, 10 mL of sodium citrate (1%) was added, and the mixture was heated until it became pale yellow. The mixture was left undisturbed while it cooled to room temperature. Then this solution was poured into the beaker containing the Mag@AC1 or Mag@AC2, and the mixture was vigorously stirred at 60 °C for 4 h using a magnetic stirrer. After that, the mixture was left undisturbed for 24 h at room temperature. Magnetite/activated carbon/silver (Mag@AC1-Ag and Mag@AC2-Ag) nanosorbents were separated by a magnet. The separated samples were then dried at 80 °C for 6 h. The prepared nanosorbents were grinded and kept for future use in sealed plastic containers.

### Characterization of magnetite, Mag@AC1-Ag, and Mag@AC2-Ag nanosorbent*s*

X-ray diffractometer (Bruker D8 Advance, Germany) was used to obtain X-ray diffraction data of the prepared nanosorbents over a 2θ range of 10–80° and Cu target (1.5406 Å). The nanosorbents’ surface morphology was described employing a scanning electron microscope (SEM Quanta FEG 250 with field emission gun, FEl Company, the Netherlands). The nanosorbents’ particle size was estimated using a high-resolution transmission electron microscope (model JEM-2100HRT, Japan) that operated at 200 kV. A Fourier-transform infrared (FTIR-6100 Jasco, Japan) spectrometer was utilized for recording the FT-IR spectra of the prepared nanosorbents in the range of 4000–400 cm^−1^ with a spectral resolution of 4 cm^−1^ at room temperature. The BET surface area and pore structure of the nanosorbents were evaluated with a Quantachrome surface area analyzer (model Autosorb-1) in an N_2_ atmosphere at 77 K using the BJH method from the desorption data. In order to get rid of moisture, the prepared nanosorbents were degassed at 200 °C for 24 h prior to measurement.

### Sorption studies

To explore the removal of Pb^+2^ and Cd^+2^ ions, batch experiments were conducted by shaking the solution of each ion (25 mL) with 0.05 g of the nanosorbent in a rotary shaker with a 180-rpm agitation speed at 25 °C. In addition, to optimize the experimental conditions for the removal of each ion, several parameters, including the initial pH (2–7), contact time (10–180 min), mass of nanosorbent (0.01–0.2 g), initial Pb^+2^ or Cd^+2^ concentration (5–400 mg L^−1^), and temperature (25, 35, 45, and 55 °C) were investigated. After reaching equilibrium, the nanosorbents were detached using a strong magnet, and the supernatant was separated using a 0.20-μm syringe filter.

The metal ions’ remaining concentration was determined using atomic absorption spectroscopy (240AAFS, Agilent, USA). The removal efficiency % of Pb^+2^ or Cd^+2^ was estimated using Eq. (1):

1 $$\mathrm{Removal\;efficiency\;\% }=\frac{\left({C}_{\mathrm{i}}-{C}_{\mathrm{e}}\right)}{{C}_{\mathrm{i}}} \times 100$$

where C_i_ and C_e_ are the initial and equilibrium Pb^+2^ or Cd^+2^concentrations, respectively, in the solution (mg L^−1^). The sorption capacity (q_e_) of the prepared nanosorbents for Pb^+2^ and Cd^+2^ was estimated using Eq. (2):

2 $${q}_{\mathrm{e}} (\mathrm{mg }\;{\mathrm{g}}^{-1}) =\frac{\left({C }_{\mathrm{i}} - {C}_{\mathrm{e}}\right)\; V }{m}$$

where the solution volume (L) is V and the nanosorbent mass (g) is m.

### Sorption kinetics

The sorption kinetics are important for describing the sorption mechanisms and rates, and it relies upon the chemical and physical features of the sorbent. For describing the sorption kinetics, Lagergren pseudo-first-order, pseudo-second-order, and intra-particle diffusion models were applied.

Lagergren pseudo-first-order model presumes that one metal ion is sorbed onto one sorption site on the sorbent surface. The equation of the Lagergren pseudo-first-order model is defined as:

3 $$\mathit{ln}\left({q}_{e}-{q}_{t}\right)=ln\;{q}_{e}-{k}_{1}t$$

where q_e_ and q_t_ (mg g^−1^) are the sorption capacities for Pb^+2^ and Cd^+2^ at equilibrium and time t (min), respectively, and k_1_ (min^−1^) is the pseudo-first-order rate constant.

On the other hand, the pseudo-second-order kinetics presumes that one metal ion is sorbed onto two sorption sites on the sorbent surface. The pseudo-second-order kinetic model is presented as:

4 $$\frac{\mathrm{t}}{{\mathrm{q}}_{\mathrm{t}}}=\frac{1}{{\mathrm{k}}_{2}{\mathrm{q}}_{\mathrm{e}}^{2}}+\frac{\mathrm{t}}{{\mathrm{q}}_{\mathrm{e}}}$$

where k_2_ is the pseudo-second-order rate constant (g mg^−1^ min^−1^).

5The Weber-Morris equation can be used to express the intra-particle diffusion model, which can be expressed as follows:

5 $${q}_{\mathrm{t}}={k}_{\mathrm{d}}\;{t}^{0.5}+C$$

where k_d_ is the intra-particle diffusion rate constant (mg g^−1^ min^−1/2^) and C is the width of the boundary layer.

In addition, the Boyd model (Pholosi et al. 2020) is employed to discriminate between film and intra-particle diffusion to determine the genuinely rate-controlling step using Eq. (6) as follows:

6 $$G=\frac{{q}_{\mathrm{t}}}{{q}_{\mathrm{e}}}=1-\frac{6}{{\uppi }^{2}}\mathrm{\;exp}\left(- {\mathrm{B}}_{\mathrm{t}}\right)$$

Rearranging Eq. (6):

7 $$\normalsize {B}_{\mathrm{t}}=-0.4977-ln\left(1-\frac{{q}_{\mathrm{t}}}{{q}_{\mathrm{e}}}\right)$$

where G denotes the fraction of Pb^+2^ or Cd^+2^ sorbed at time t and the mathematical function of G is B_t_. The estimated B_t_ values using Eq. (7) were plotted against t.

### Sorption isotherms

Sorption isotherms illustrate the mathematical correlation between the sorbate quantity and the sorbate concentration at equilibrium that still present in the solution at a fixed temperature. The equilibrium sorption data has been explored using Langmuir, Freundlich, Dubinin–Radushkevich, and Temkin isotherms (Abatal et al. 2021)**.**

The Langmuir isotherm linearized form can be represented by:

8 $$\frac{{C}_{e}}{{q}_{e}}=\frac{1}{{K}_{\mathrm{L}}{\mathrm{q}}_{\mathrm{max}}} +\frac{{\mathrm{C}}_{\mathrm{e}}}{{q}_{\mathrm{max}}}$$

where q_max_ (mg g^−1^) is the maximum Langmuir sorption capacity and the Langmuir constant is K_L_ (L mg^−1^), which is related to binding site affinity and sorption bonding energy.

Freundlich isotherm is represented by:

9 $$log\;{q}_{\mathrm{e}} =\frac{1}{\mathrm{n}}log\;{C}_{\mathrm{e}} + log\;{K}_{\mathrm{F}}$$

where the Freundlich constant is K_F_ (mg g^−1^), which represents sorption capacity, and n (L mg^−1^) is the sorption intensity.

Dubinin–Radushkevich isotherm (D-R) model can be expressed by:

10 $$ln{q}_{e}=ln {K}_{\mathrm{D}-\mathrm{R}}-{\upbeta \varepsilon }^{2}$$

where K_D-R_ (mg g^−1^) is the D-R constant and ε (mol^2^ J^−2^) is the Polanyi potential, which is equal to:

11 $$\varepsilon =RT\;ln\;\left(1+\frac{1}{{C}_{e}}\right)$$

where the ideal gas constant is R (J K^−1^ mol^−1^), and the absolute temperature is T (K). The mean sorption free energy, E (kJ mol^−1^), is associated with the constant β and is represented by:

12 $$E=1/\sqrt{2\beta }$$

The Temkin isotherm’s linearized form is represented as follows:

13 $${q}_{e} = RT/{b}_{T}\; ln \;{A}_{T} + RT/ {b}_{T} \; ln \;{C}_{e}$$

where A_T_ is a constant equivalent to the maximum binding energy (L g^−1^) and *b*_T_ (J mol^−1^) is the Temkin isotherm constant associated with sorption heat.

### Thermodynamics studies

The impact of temperature on the Pb^2+^ and Cd^2+^ sorption using magnetite, Mag@AC1-Ag, and Mag@AC2-Ag nanosorbents was investigated by performing experiments using 100 and 50 mg L^−1^ of initial Pb^+2^ and Cd^+2^ concentrations, respectively, at 298, 308, 318, and 328 K. The thermodynamic parameters, Gibbs free energy change (Δ*G*), enthalpy change (Δ*H*), and entropy change (Δ*S*), were evaluated using the following equations:

14 $$\Delta G=\Delta H-T\Delta S$$

15 $${K}_{\mathrm{d}} = \frac{{q}_{\mathrm{e}}}{{C}_{\mathrm{e}}}$$

16 $$\mathit{ln}\;{K}_{\mathrm{d}}= \frac{\Delta S}{R} - \frac{\Delta H}{RT}$$

### Regeneration and reusability studies

The probability of regeneration and reuse of sorbents is of environmental and economic concerns. For regeneration experiments, Pb- or Cd-loaded nanosorbent was first rinsed with distilled water several times to remove any metal ions that were not firmly attached to their surface. The desorption procedure was carried out by mixing 0.05 g of Pb- and Cd-loaded nanosorbents with 10 mL of HCl (0.1 mol L^−1^) for an hour on a rotary shaker at an agitation speed of 180 rpm at 25 °C. Desorption efficiency was estimated by Eq. (17):

17 $$\mathrm{Desorption\;efficiency\;\% }= \frac{\mathrm{Amount\;of\;released\;metal\;ion\;}}{\mathrm{Amount\;of\;sorbed\;metal\;ion }} \times 100$$

For the reusability process, the regenerated nanosorbents were tested by following the sorption–desorption procedure for several cycles for both Pb^+2^ and Cd^+2^ removal.

### Nanosorbents’ antimicrobial activity

The antimicrobial activity of nanosorbents against pathogenic bacteria, including fecal coliform (gram-negative) and *Bacillus subtilis* (gram-positive), was tested utilizing the agar well diffusion method. On a nutrient agar medium, bacteria pure cultures were subcultured. Each strain was swabbed with sterile cotton swabs onto plates. A paper disc comprising the nanosorbent solution was then placed on the agar surface. After leaving the Petri dishes for 24 h at 37 °C, the width of the inhibition zone surrounding each disc was evaluated.

## Results and discussion

### Characterization

#### Point of zero charge (pH_pzc_)

**Fig. 1:**
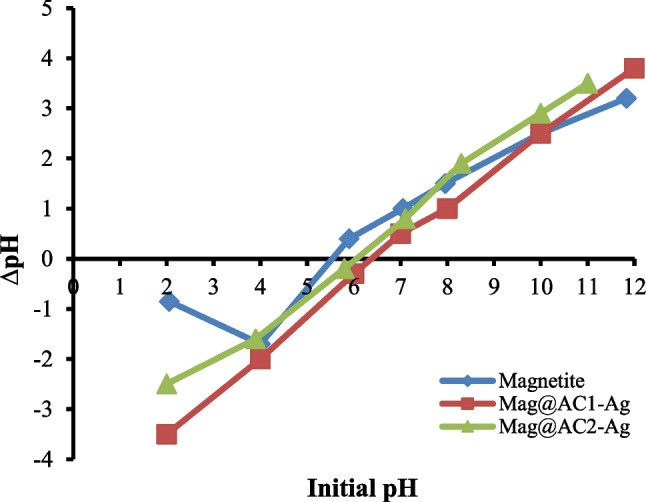
pH_zpc_ for magnetite, Mag@AC1-Ag, and Mag@AC2-Ag

The pH_pzc_ of the prepared nanosorbents was estimated using the batch equilibrium method (Jain et al. 2018). The initial pH (pH_i_) of 50 mL of NaCl solutions (0.01 mol L^−1^) was set between 2 and 12 with HCl or NaOH (0.1 mol L^−1^) where 0.1 g of magnetite, Mag@AC1-Ag, or Mag@AC2-Ag was added. The flasks containing nanosorbents were agitated for 48 h, and the final pH (pH_f_) of the solution was estimated. The difference between the pH_i_ and pH_f_ was plotted versus the pH_i_. pH_pzc_ is the value obtained at the point where the resultant curve intersects the abscissa axis, with ∆pH equals 0. The obtained pH_pzc_ values of magnetite, Mag@AC1-Ag, and Mag@AC2-Ag (Fig. 1) were found to be 5.6, 6.4, and 6.0, respectively.

#### X-ray diffraction (XRD)

**Fig. 2:**
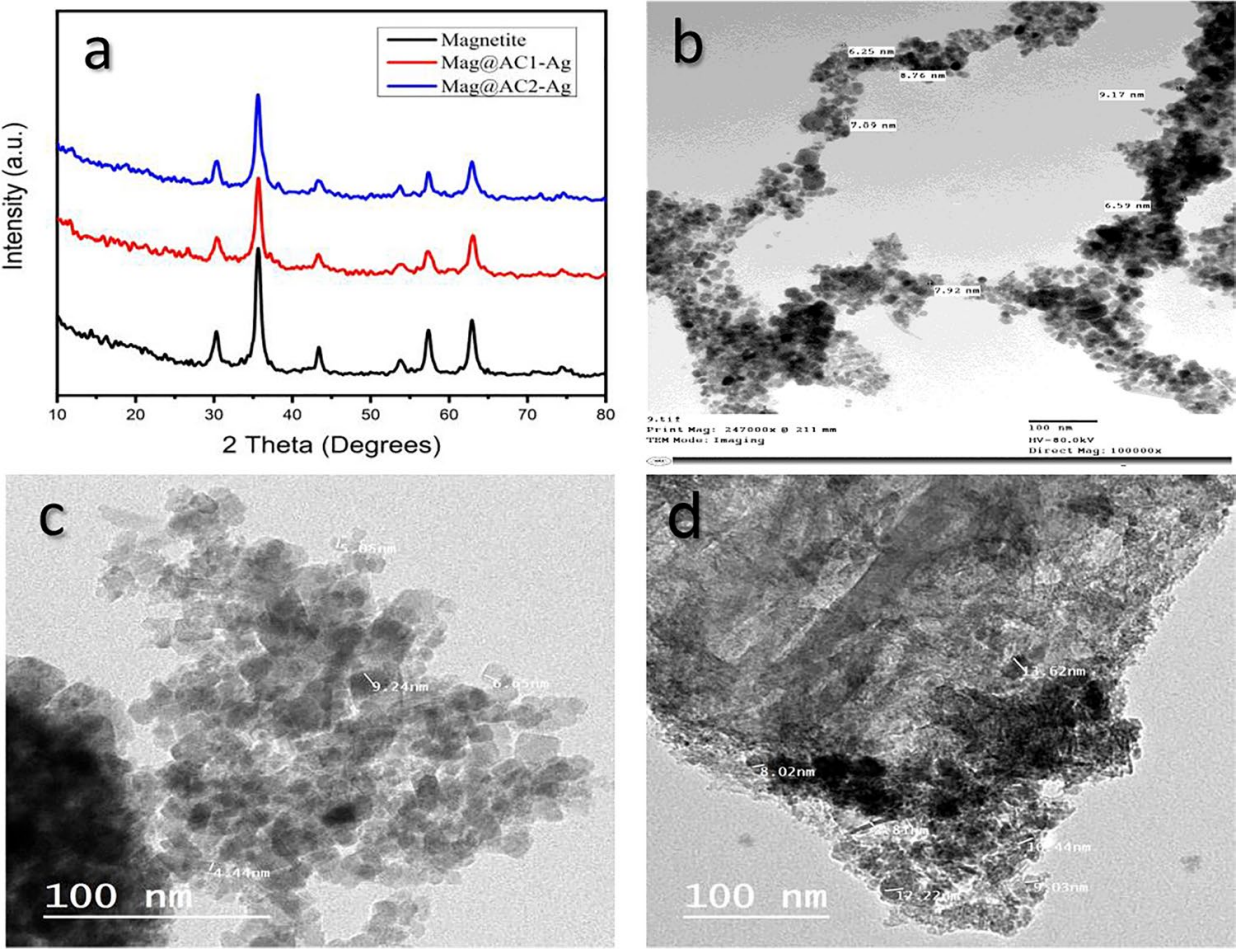
XRD patterns of magnetite, Mag@AC1-Ag, and Mag@AC2-Ag (**a**) and TEM images of magnetite (**b**), Mag@AC1-Ag (**c**), and Mag@AC2-Ag (**d**)

The XRD patterns of magnetite, Mag@AC1-Ag, and Mag@AC2-Ag are shown in Fig. 2a. The typical peak at 35.65° present in all three samples is attributed to the crystalline plane with a Miller index of (3 1 1), which proves the inverse spinal structure of magnetite (Bastami and Entezari 2012). The characteristic diffraction peaks at 2θ of 30.27°, 43.33°, 53.77°, 57.32°, 62.95°, 67.26°, 71.43°, and 74.50° are indexed to (2 2 0), (4 0 0), (4 2 2), (5 1 1), (4 4 0), (4 4 2), (6 2 0), and (5 3 3) planes, respectively, suggesting the presence of a cubic magnetite phase (JCPDS Card no. 79–0417). In the case of Mag@AC1-Ag and Mag@AC2-Ag samples, the characteristic peaks of magnetite are still present but with less intensity, demonstrating the successful incorporation of magnetite within the activated carbon matrix. Also, weak characteristic peaks of AgNPs were presented at 2θ of 38.17°, 44.53°, and 64.44° indexed to (1 1 1), (2 0 0), and (2 2 0), planes, respectively, and this may be due to the low content of Ag nanoparticles. The XRD results for Mag@AC1-Ag and Mag@AC2-Ag samples confirm that magnetite and Ag nanoparticles have been successfully incorporated into the activated carbon matrix. The estimated crystallite size was 11.83 nm for magnetite, 13.51 nm for Mag@AC1-Ag, and 10.54 nm for Mag@AC2-Ag, all obtained using Scherrer’s formula (Cullity 1956).

#### Transmission electron microscopy (TEM)

Figure 2b–c displays the TEM images for magnetite, Mag@AC1-Ag, and Mag@AC2-Ag samples. The figure indicates that the nanosorbents have a pseudo-spherical shape. Moreover, the figure shows that the particle sizes of magnetite, Mag@AC1-Ag, and Mag@AC2-Ag nanosorbents were in the range of 6–10, 4–10, and 8–14 nm, respectively.

#### Scanning electron microscopy (SEM)/energy dispersive X-ray spectroscopy (EDX)

**Fig. 3:**
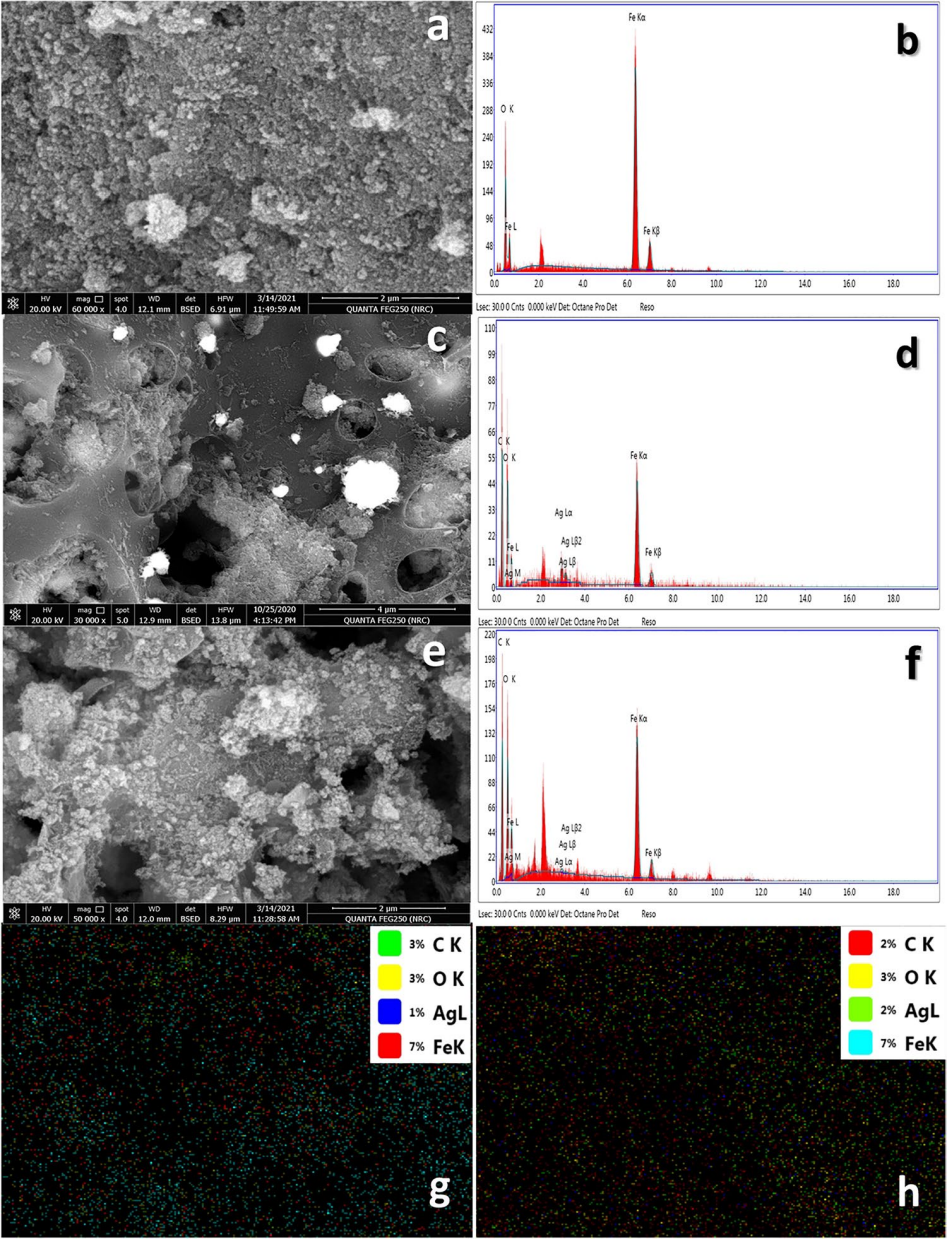
SEM images and EDX survey for magnetite (**a**, **b**), Mag@AC1-Ag (**c**, **d**), and Mag@AC2-Ag (**e**, **f**), respectively, and elemental mapping for Mag@AC1-Ag (**g**) and Mag@AC2-Ag (**h**)

SEM images and EDX survey are important for describing the surface morphology, physical properties, and structure of the sorbents. Figure 3 shows SEM images and EDX spectra of the prepared magnetite, Mag@AC1-Ag, and Mag@AC2-Ag nanosorbents. As revealed from the images, the magnetite nanoparticles (Fig. 3a) are agglomerated, stuck to each other, and have a spongy-like texture. Figure 3c, e shows that both Mag@AC1-Ag and Mag@AC2-Ag have porous, rough, and coarse surfaces. It can also be observed that the surfaces of both Mag@AC1-Ag and Mag@AC2-Ag samples are shiny and bright due to the precipitation of silver nanoparticles. The EDX elemental analysis shown in Fig. 3b, d, f displays major peaks of C, O, Fe, and Ag. These major peaks confirm the incorporation of magnetite and silver nanoparticles within the activated carbon matrix. In addition, a homogenous dispersion of Fe and Ag can be seen on the surface of activated carbon for Mag@AC1-Ag and Mag@AC2-Ag samples (Fig. 3g, h).

#### Fourier transform infrared spectroscopy (FT-IR)

**Fig. 4:**
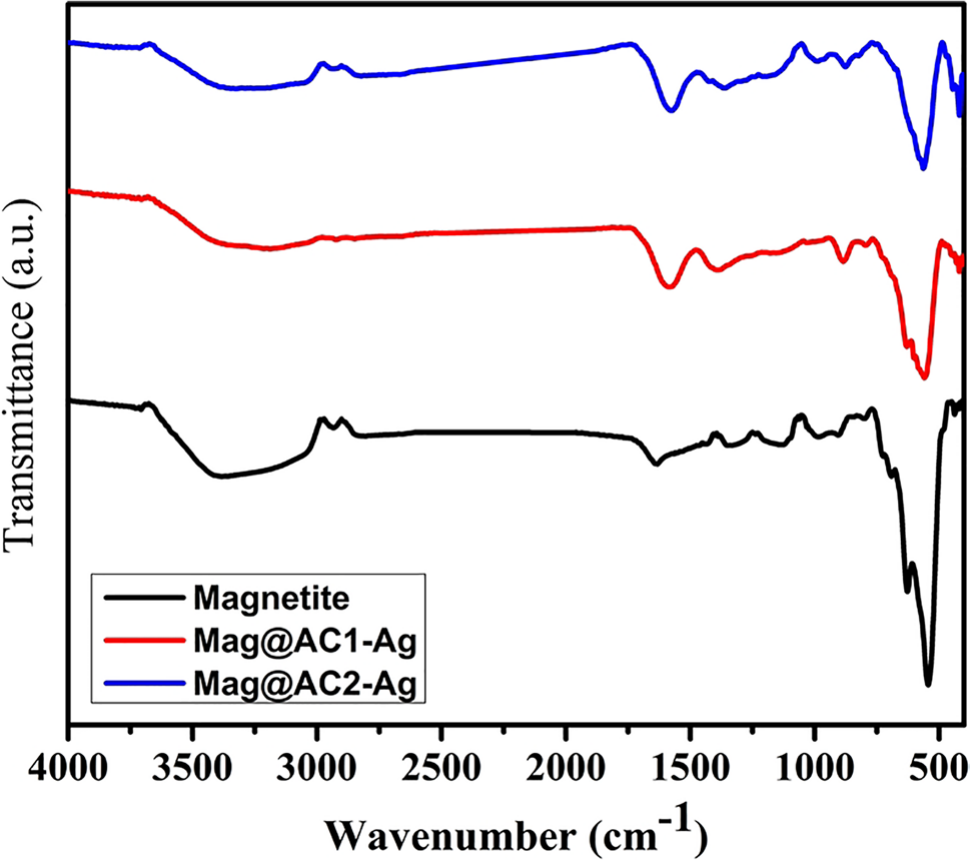
FT-IR spectra of magnetite, Mag@AC1-Ag, and Mag@AC2-Ag

The FT-IR spectra of magnetite, Mag@AC1-Ag, and Mag@AC2-Ag samples are shown in Fig. 4. The wide peaks at around 3382, 3182, and 3212 cm^−1^ indicate the O–H stretching vibration mode of the hydroxyl groups. The peaks of O–H stretching vibrations are broader in the cases of Mag@AC1-Ag and Mag@AC2-Ag due to the carboxyl groups of activated carbon. As illustrated in the figure, the peaks at around 2927 and 2920 cm^−1^ are the C-H stretching vibration of the CH_3_ groups in Mag@AC1-Ag and Mag@AC2-Ag, respectively, while the peaks at 1574 and 1568 cm^−1^ indicate the C = O stretching vibration of carboxyl/the aromatic ring C = C bond (Marzbani et al. 2016; Cai et al. 2021). In the IR spectra of Mag@AC1-Ag and Mag@AC2-Ag, the absorption peaks at 1381 and 1392 cm^−1^, respectively, are assigned to symmetric stretching of -COO (Torab-Mostaedi et al. 2013; Li et al. 2017). Moreover, the presence of absorption peaks at about 544, 553, and 553 cm^−1^ in magnetite, Mag@AC1-Ag, and Mag@AC2-Ag, respectively, is assigned to the characteristic Fe–O stretching vibration, implying the existence of magnetite nanoparticles on the nanosorbents’ surface (Jain et al. 2018).

#### Surface area and particle size analysis

**Fig. 5:**
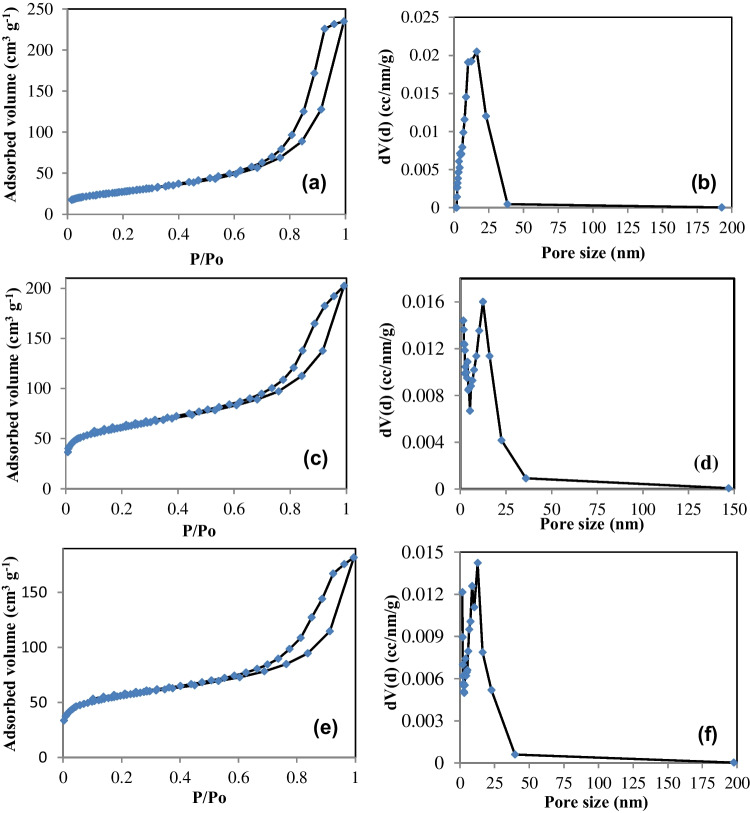
N_2_ adsorption–desorption isotherms and particle size distribution of magnetite (**a**, **b**), Mag@AC1-Ag (**c**, **d**), and Mag@AC2-Ag (**e**, **f**), respectively

The N_2_ adsorption/desorption isotherms were determined for the prepared samples and are given in Figure S1 and Fig. 5. The samples of AC1 and AC2 exhibit type I isotherm (Figure S1), demonstrating that AC1 and AC2 have microporous structures (Li et al. 2014). On the other hand, magnetite, Mag@AC1-Ag, and Mag@AC2-Ag exhibit type IV isotherm, indicating the formation of mesoporous structures with a H1 hysteresis loop (Asghar et al. 2017). The hysteresis loop exists in the high relative region (P/P_o_ > 0.6) for magnetite and in P/P_o_ > 0.50 for Mag@AC1-Ag and Mag@AC2-Ag. At the lower pressure regions, the samples show the formation of a monolayer followed by the formation of multilayers. As shown in Fig. 5c, e, the amount of N_2_ that has been adsorbed in the cases of Mag@AC1-Ag and Mag@AC2-Ag was low. It may be because magnetite and/or Ag nanoparticles covered the surface and filled the pores of the activated carbon. According to Fig. S1(b, d), AC1 and AC2 samples possessed internal pores that were predominantly micropores with a considerable amount of mesopores. Also, the results shown in Fig. 5b, d, and f indicated that magnetite, Mag@AC1-Ag, and Mag@AC2-Ag had pores inside, which were mainly mesopores. Therefore, the prepared nanosorbents may have excellent sorption performance.

The estimated BET surface areas, average pore diameters, and total pore volumes are listed in Table 1. As seen, the surface area of AC1 and AC2 decreased upon magnetite and silver nanoparticles incorporation in their structures. This may be due to some magnetite, or/and Ag nanoparticles have blocked the pores of activated carbon. Furthermore, it can be observed that Mag@AC1-Ag and Mag@AC2-Ag had high specific surface areas and rich pore structures, as revealed from SEM images as well. These findings suggest that Mag@AC1-Ag and Mag@AC2-Ag can be efficient sorbents for a variety of contaminants.

**Table 1:** Calculated total specific surface area (S_BET_), total pore volume (V_tot_), and average pore diameter (D_p_)

Sample	S_BET_ (m^2^ g^−1^)	V_tot_ (cm^3^ g^−1^)	D_P_ (nm)
AC1	426.95	0.21	1.96
AC2	364.39	0.19	2.04
Magnetite	97.53	0.36	14.91
Mag@AC1-Ag	220.9	0.31	5.67
Mag@AC2-Ag	203.6	0.28	5.52

### Batch sorption studies

#### Impact of initial pH

**Fig. 6:**
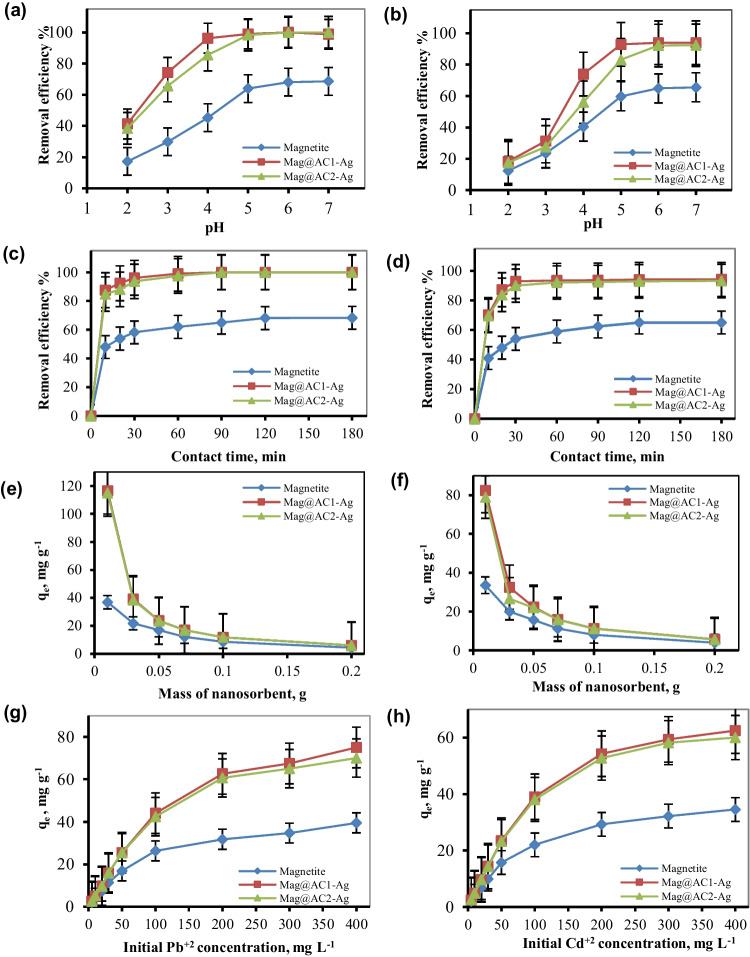
Impact of initial pH, contact time, mass of nanosorbent, and initial metal concentration on the removal of Pb^+2^ (**a**, **c**, **e**, **g**) and Cd^+2^ (**b**, **d**, **f**, **h**) using magnetite, Mag@AC1-Ag, and Mag@AC2-Ag

The impact of initial pH on the sorption of Pb^2+^ and Cd^2+^ ions by magnetite, Mag@AC1-Ag, and Mag@AC2-Ag was studied in the range of 2–7 to avoid Pb^2+^ and Cd^2+^ ion precipitation above pH > 7. The results are presented in Fig. 6a, b. As shown in the figure, the removal of Pb^2+^ and Cd^2+^ was improved by increasing the initial pH of the metal ion solution. At lower pH values, Pb^2+^ and Cd^2+^ ion removal decreased due to the increased H^+^ ion competition for active sites. It would be suggested that at lower pH values, the nanosorbent surface is surrounded by H^+^, and the surface becomes more positively charged, reducing the attraction between nanosorbents and metal ions. In contrast, when the pH increased, the functional groups on the surface of the nanosorbents, such as carboxylic acid groups, were deprotonated. Consequently, more negatively charged surfaces were created, leading to an electrostatic attraction between the positively charged metal ions and these negatively charged sorption sites; hence, their removal was increased. Based on these observations, in addition to the determined pHpzc values (section “Point of zero charge (pH_pzc_)”), it can be concluded that Pb^2+^ and Cd^2+^ removal might take place via several sorption processes (electrostatic attraction, surface complexation, ion-exchange, and coprecipitation). The same observation was also reported for the removal of Pb^2+^ and Cd^2+^ using mango seed biosorbent (Wang et al. 2022).

#### Impact of contact time

Pb^2+^ and Cd^2+^ ion removal was investigated at various time intervals of 10, 20, 30, 60, 90, 120, and 180 min. The other parameters, such as the initial Pb^2+^ or Cd^2+^ concentration (50 mg L^−1^), nanosorbent mass (2 g L^−1^), and temperature (25 °C) were kept constant. As revealed from Fig. 6c, d, the removal efficiency of Pb^2+^ and Cd^2+^ improved with increasing contact time until equilibrium was accomplished. The results demonstrated that the sorption process was very fast, where the prepared nanosorbents (Mag@AC1-Ag and Mag@AC2-Ag) showed rapid metal ion reduction in the first 10 min of contact time, followed by a progressive rise until equilibrium. The sorption onto Mag@AC1-Ag and Mag@AC2-Ag reached equilibrium in 60 min, after which no major change in the Pb^2+^ and Cd^2+^ removal percentage occurred. At the initial stages, the greater number of available sites on the nanosorbents’ surfaces may have contributed to the higher sorption rates of Pb^2+^ and Cd^2+^. The succeeding slower sorption was probably due to the contending among the Pb^2+^ or Cd^2+^ ions for the few available unsaturated sorption sites. As the nanosorbents’ surfaces were saturated, the diffusion of Pb^2+^ or Cd^2+^ into the nanosorbents’ bulk slowed down and the sorption rate diminished. To ensure complete sorption of Pb^2+^ and Cd^2+^ ions from the solution, the experiments were conducted for 120 min.

To check the stability of the prepared nanosorbents, the amounts of nanoparticles that might be released from the surface of activated carbon after equilibrium were measured. The results revealed that the amount of Ag ions released was between 0.32 and 0.35 mg L^−1^) and that of Fe ions was between 0.095 and 0.92 mg L^−1^), indicating the stability of the prepared nanosorbents.

#### Impact of nanosorbent mass

The effluence of nanosorbent mass on the Pb^+2^ and Cd^+2^ removal using magnetite, Mag@AC1-Ag, and Mag@AC2-Ag is shown in Fig. 6e, f. As observed, the sorption capacities diminished with an increase in the mass of nanosorbents. This may be due to particle interactions like aggregation driven on by the high nanosorbent mass, which result in a decrease in the nanosorbent’s active surface area (Kakavandi et al. 2015).

#### Impact of Pb^+2^ or Cd^+2^ concentration

Figure 5g, h depict the impact of Pb^+2^ and Cd^+2^ concentrations on their removal by magnetite, Mag@AC1-0, and Mag@AC2-Ag at concentrations ranging from 5 to 400 mg L^−1^, while all other process variables are held constant. The findings demonstrated that the sorption capacity increased along with the initial Pb^+2^ and Cd^+2^ concentrations. The sorption capacities raised from 2.13 to 39.51, 2.51 to 75, and 2.51 to 70 mg g^−1^ for Pb^+2^ by magnetite, Mag@AC1-Ag, and Mag@AC2-Ag, respectively. On the other hand, Cd^+2^ sorption capacities increased from 2.05 to 34.5, 2.46 to 62.5, and 2.45 to 60 mg g^−1^ for magnetite, Mag@AC1-Ag, and Mag@AC2-Ag, respectively. It can be noted that the Mag@AC1-Ag nanosorbent has a better sorption capacity towards Pb^+2^ and Cd^+2^ than the Mag@AC2-Ag sample. This can be attributed to its larger surface area, as revealed from BET analysis data. As the initial Pb^+2^ and Cd^+2^ concentrations rise, the mass transfer driving force overwhelms the barrier to Pb^+2^ and Cd^+2^ mass transfer from the solution to the nanosorbents. This causes greater contact between the nanosorbents’ surface and Pb^+2^ and Cd^+2^, hence increasing the sorption capacity. The preference of the nanosorbents for Pb^+2^ ions on the prepared nanosorbents is due to the fact that Pb has a smaller hydrated ionic radius than Cd (0.401 nm vs. 0.426 nm) (Yu et al. 2013).

### Sorption kinetics

The sorption kinetics of Pb^+2^ and Cd^+2^ removal using magnetite, Mag@AC1-Ag, and Mag@AC2-Ag were elucidated using Lagergren pseudo-first-order, pseudo-second-order, and intra-particle diffusion models, and Table 2 lists the key parameters for each model.

**Table 2:** Calculated parameters of the pseudo-first-order, pseudo-second-order, intra-particle diffusion, and Boyd models for Pb^+2^ and Cd^+2^ removal using magnetite, Mag@AC1-Ag, and Mag@AC2-Ag

Model	Metal ion	Parameters	Nanosorbent		
			Magnetite	Mag@AC1-Ag	Mag@AC2-Ag
Pseudo-first-order	Pb^+2^	k_1_	0.022	0.049	0.047
		q_e, calc._ (mg g^−1^)	5.65	4.98	5.80
		q_e, exp._ (mg g^−1^)	16.72	25.46	25.46
		R^2^	0.9527	0.9937	0.9703
	Cd^+2^	k_1_	0.043	0.051	0.040
		q_e, calc._ (mg g^−1^)	11.36	4.43	3.94
		q_e, exp._ (mg g^−1^)	15.30	21.90	21.66
		R^2^	0.8845	0.9239	0.9268
Pseudo-second-order	Pb^+2^	k_2_	0.009	0.027	0.016
		q_e, calc._ (mg g^−1^)	17.54	25.70	25.90
		q_e, exp._ (mg g^−1^)	16.72	25.46	25.46
		R^2^	0.9994	1.0000	0.9999
	Cd^+2^	k_2_	0.008	0.024	0.019
		q_e, calc._ (mg g^−1^)	16.13	22.22	22.03
		q_e, exp._ (mg g^−1^)	15.30	21.90	21.66
		R^2^	0.9996	0.9998	0.9999
Intra-particle diffusion	Pb^+2^	k_d_	1.083	0.938	0.942
		C	8.37	19.33	18.42
		R^2^	0.9998	1	0.8841
	Cd^+2^	k_d_	1.317	2.304	2.079
		C	5.45	9.33	9.77
		R^2^	0.9990	0.9583	0.9788
Boyd model	Pb^+2^	R^2^	0.9999	0.9933	0.8854
	Cd^+2^	R^2^	0.9965	0.9930	0.9976

#### Lagergren pseudo-first-order kinetics

As shown in Table 2, the estimated q_e_ values are not close to the experimental q_e_ values, revealing that the pseudo-first-order model (Figures S2 and S3) was not proper to explain the Pb^+2^ and Cd^+2^ sorption kinetics onto magnetite, Mag@AC1-Ag, and Mag@AC2-Ag nanosorbents.

#### Pseudo-second-order kinetics

The pseudo-second-order kinetics’ results are shown in Figures S4 and S5, which illustrate the relationship between t/q_t_ and t for the Pb^2+^ and Cd^+2^ removal using magnetite, Mag@AC1-Ag, and Mag@AC2-Ag. It is evident that a straight line is used to depict the relationship. Table 2 also shows that the regression coefficient values are close to unity for Pb^2+^ and Cd^2+^ for all nanosorbents, and the estimated q_e_ values are very similar to those of the experimentally obtained q_e_, revealing that the pseudo-second-order model is more appropriate to describe the kinetics of Pb^2+^ and Cd^2+^ sorption onto all nanosorbents.

#### Intra-particle diffusion model

**Fig. 7:**
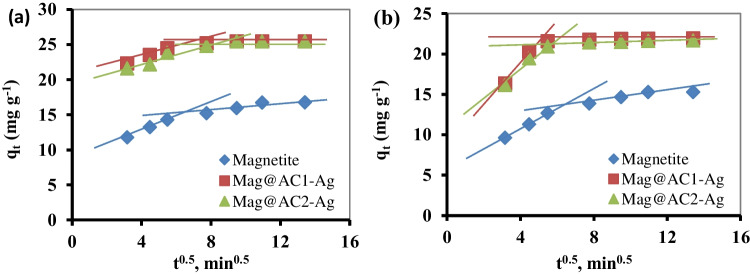
Intra-particle diffusion plots for the removal of Pb^+2^ (**a**) and Cd^+2^ (**b**) using magnetite, Mag@AC1-Ag, and Mag@AC2-Ag

The plots of the intra-particle diffusion model are shown in Fig. 7a, b. The plots are shown in Fig. 7a, b. The figure shows the presence of two stages during the Pb^+2^ and Cd^+2^ sorption onto magnetite, Mag@AC1-Ag, and Mag@AC2-Ag. The first stage represents the film diffusion ascribed to the external mass transport of Pb^+2^ and Cd^+2^ ions from the bulk solution to the nanosorbent surface. The second stage was related to the Pb^+2^ and Cd^+2^ ions’ diffusion through the pores of nanosorbents, which became sluggish due to the decrease in the metal ions’ concentration or the decrease of effective sorption sites on the nanosorbents (Jiang et al. 2018). If intra-particle diffusion is the only rate-limiting step, the graph of q_t_ vs. t^0.5^ will be linear, and the line will intersect the origin. However, in the present case, the lines did not intersect at the origin, which indicated that the rate-limiting step of the sorption of Pb^+2^ and Cd^+2^ was simultaneously controlled by film and intra-particle diffusion (Hu et al. 2020).

#### Boyd model

It is clear from the intra-particle diffusion investigation that the process of Pb^+2^ and Cd^+2^ removal using magnetite, Mag@AC1-Ag, and Mag@AC2-Ag was a multi-step process. However, there is still a consideration regarding the step that governs the overall rate of sorption. For that reason, the mathematical expression of the Boyd model was applied to the sorption data in order to define the actual rate-limiting step. The characteristics of Boyd plots provided essential data about whether film or intra-particle diffusion identified the rate-limiting step. When the obtained plot is linear and traverses the origin, intra-particle diffusion is thought to be the rate-limiting mechanism. Nevertheless, if the plot does not traverse the origin, external mass transfer (film diffusion) limits the overall rate of the Pb^+2^ and Cd^+2^ removal process. As shown in Figures S6 and S7, the plots were linear at the initial stage of sorption (R^2^ is illustrated in Table 2). The obtained plots for the Boyd model did not traverse the origin, revealing that the Pb^+2^ and Cd^+2^ sorption onto the prepared nanosorbents at the beginning of the sorption process was regulated by film diffusion (Ofomaja 2010).

### Sorption isotherms

Different isotherms were used to express the relationship between Pb^+2^ and Cd^+2^ ions’ equilibrium concentrations in the solid and liquid phases, including the Langmuir, Freundlich, D-R, and Temkin isotherms.

#### Langmuir isotherm

The Langmuir isotherm presumes that a sorbent contains a limited number of binding sites of identical energy. One molecule is sorbed by each site, resulting in a single monolayer. Figures S8 and S9 show the plots of C_e_/q_e_ vs. C_e_, whereas the values of q_max_, k_L_, and R^2^ are listed in Table 3. As observed, the q_max_ values were 41.67, 72.99, and 68.97 mg g^−1^ for Pb^+2^ and 37.04, 62.50, and 60.61 mg g^−1^ for Cd^+2^ using magnetite, Mag@AC1-Ag, and Mag@AC2-Ag, respectively. The sorption capacities of the prepared nanosorbents followed the order: Mag@AC1-Ag > Mag@AC2-Ag > magnetite.

**Table 3:** Parameters of the Langmuir, Freundlich, Dubinin-Radushkevich (D–R), and Temkin isotherm models for the sorption of Pb^+2^ and Cd^+2^ onto magnetite, Mag@AC1-Ag, and Mag@AC2-Ag

Isotherm model	Metal ion	Parameters	Nanosorbent		
			Magnetite	Mag@AC1-Ag	Mag@AC2-Ag
Langmuir	Pb^+2^	K_L_ (L mg^−1^)	0.044	0.320	0.340
		q_max_ (mg g^−1^)	41.67	72.99	68.97
		R^2^	0.9925	0.9946	0.9964
		*R*_L_	0.3160	0.0076	0.0073
	Cd^+2^	K_L_ (L mg^−1^)	0.041	0.150	0.165
		q_max_ (mg g^−1^)	37.04	62.50	60.61
		*R*^2^	0.9964	0.9974	0.9979
		*R*_L_	0.336	0.110	0.118
Freundlich	Pb^+2^	K_F_	12.03	28.11	26.10
		1/n	0.201	0.177	0.181
		R^2^	0.9658	0.9856	0.9732
	Cd^+2^	K_F_	2.85	10.05	9.33
		1/n	0.475	0.374	0.381
		R^2^	0.9542	0.9371	0.9364
Dubinin-Radushkevich (D–R)	Pb^+2^	K_D-R_	35.94	68.70	65.74
		β*10^−3^	0.114	0.014	0.021
		E (kJ mol^−1^)	2.09	5.94	4.87
		R^2^	0.7845	0.9113	0.9465
	Cd^+2^	K_D-R_	33.11	59.44	57.77
		β*10^−3^	0.195	0.038	0.044
		E (kJ mol^−1^)	1.60	3.63	3.37
		R^2^	0.9483	0.9485	0.9567
Temkin	Pb^+2^	b_T_ (kJ mol^−1^)	0.387	0.244	0.252
		A_T_	1.19	5.82	4.84
		R^2^	0.9386	0.9867	0.9854
	Cd^+2^	b_T_ (kJ mol^−1^)	0.412	0.312	0.314
		A_T_	0.85	8.13	6.68
		R^2^	0.9775	0.9826	0.9842

To assess the feasibility and favorability of the Pb^+2^ and Cd^+2^ sorption, the separation factor (*R*_L_) was estimated as follows:

18 $${R}_{\mathrm{L}} =\frac{1}{1+{\mathrm{K}}_{\mathrm{L}}{\mathrm{C}}_{\mathrm{i}}}$$

*R*_L_ value specifies whether the isotherm is unfavorable (*R*_L_ > 1), favorable (0 < *R*_L_ < 1), linear (*R*_L_ = 1), or irreversible (*R*_L_ = 0). As displayed in Table 3, the *R*_L_ values were between 0 and 1, indicating a favorable Pb^+2^ and Cd^+2^ sorption process onto magnetite, Mag@AC1-Ag, and Mag@AC2-Ag.

#### Freundlich isotherm

According to the Freundlich isotherm, sorbents have heterogeneous surfaces with various sorption potential locations. Additionally, it presumes that stronger binding sites are firstly occupied and that binding strength diminishes as occupancy increases. The plots of log q_e_ vs. log C_e_ are displayed in Figures S10 and S11, whereas n and K_F_ values are listed in Table 3. In the case of Mag@AC1-Ag, the higher values of K_F_ than those of magnetite and Mag@AC2-Ag indicated the high sorption efficacy of Mag@AC1-Ag. The value of 1/n quantifies how heterogeneous a surface is, where the closer the value of 1/n is to zero, the more heterogeneous the surface. According to the table, 1/n values vary from 0 to 1, which reflects the magnetite, Mag@AC1-Ag, and Mag@AC2-Ag surfaces’ heterogeneity.

As can be noticed from the table, the values of (R^2^) for the Langmuir model were higher than those for the Freundlich model, thus implying that the Langmuir model better suited the experimental sorption data, thereby indicating monolayer sorption. This also suggests that the surfaces of the prepared nanosorbents are homogeneous in nature.

#### Dubinin–Radushkevich isotherm (D-R)

The D-R model discriminates between chemical and physical sorption processes. The obtained plots of ln q_e_ vs. *ε*^2^ are displayed in Figures S12 and S13. The value of E (Table 3) reveals the sorption mechanism, which is either physisorption if E is less than 8 kJ mol^−1^ or chemisorption if E is between 8 and 16 kJ mol^−1^. The values of E in this study were less than 8 kJ mol^−1^, indicating that the sorption process of Pb^+2^ and Cd^+2^ onto magnetite, Mag@AC1-Ag, and Mag@AC2-Ag may have been dominated by physisorption.

#### Temkin isotherm

According to the Temkin isotherm, interactions between the sorbent and the sorbate cause the sorption energy to decrease linearly with surface coverage. The values of *A*_T_ and b_T_ can be estimated from the q_e_ vs. ln C_e_ plot (Figures S14 and S15). As given in Table 3, the values of b_T_ ranged from 0.189 to 0.387 kJ mol^−1^. Weak electrostatic interactions can be distinguished by b_T_ values that are less than 20 kJ mol^−1^. Therefore, these results further emphasized that the sorption of Pb^+2^ and Cd^+2^ onto magnetite, Mag@AC1-Ag, and Mag@AC2-Ag showed good compatibility with the physisorption process (Mohammadnezhad et al. 2017).

### Thermodynamics studies

The temperature impact on the Pb^2+^ and Cd^2+^ sorption onto the prepared nanosorbents was evaluated. With rising temperature, it was observed that the percentage of metal ions removed increased. This revealed that the Pb^2+^ and Cd^2+^sorption process onto magnetite, Mag@AC1-Ag, and Mag@AC2-Ag was endothermic in nature. The plots of ln K_d_ vs. 1/T are displayed in Figures S16 and S17. The values of the thermodynamic parameters are tabulated in Table 4. The negative Δ*G* values for the sorption onto Mag@AC1-Ag and Mag@AC2-Ag reflected the spontaneous nature of the sorption process. However, in the case of magnetite, ΔG is positive, demonstrating that the Pb^+2^ and Cd^+2^ sorption is unfavorable. As noticed, the absolute values of Δ*G* raised as the temperature rose, implying that the high temperatures were preferred for the sorption of Pb^+2^ and Cd^+2^ onto Mag@AC1-Ag and Mag@AC2-Ag. The positive values of Δ*H* revealed the endothermic character of the sorption process. As noticed in Table 4, the Δ*H* values were between 38.99 and 65.71 kJ mol^−1^ for Mag@AC1-Ag and Mag@AC2-Ag, demonstrating a physico-chemical sorption process of the studied metals onto these nanosorbents (Liu and Liu 2008). On the other hand, the Δ*H* values for magnetite were less than 20 kJ mol^−1^, denoting a physisorption process (Liu and Liu 2008). Furthermore, the positive Δ*S* values suggested that the randomness raised at the solid/liquid interface during the Pb^+2^ and Cd^+2^ sorption onto magnetite, Mag@AC1-Ag, and Mag@AC2-Ag.

**Table 4:** Thermodynamic parameters for sorption of Pb^+2^ and Cd^+2^ onto magnetite, Mag@AC1-Ag, and Mag@AC2-Ag

Metal ion	Nanosorbent	∆*H* (kJ mol^−1^)	∆*S* (J mol^−1^ K^−1^)	∆*G* (kJ mol^−1^)				R^2^
				298 K	308 K	318 K	328 K	
Pb^+2^	Magnetite	7.35	20.22	1.325	1.123	0.921	0.718	0.9258
	Mag@AC1-Ag	65.71	228.84	− 2.483	− 4.771	− 7.060	− 9.348	0.9200
	Mag@AC2-Ag	58.39	202.46	− 1.945	− 3.970	− 5.994	− 8.019	0.9542
Cd^+2^	Magnetite	6.01	19.67	0.152	− 0.045	− 0.241	− 0.439	0.9939
	Mag@AC1-Ag	45.31	169.33	− 5.152	− 6.846	− 8.539	− 10.232	0.9833
	Mag@AC2-Ag	38.99	146.86	− 4.774	− 6.243	− 7.711	− 9.180	0.9783

### Desorption and reusability studies

**Fig. 8:**
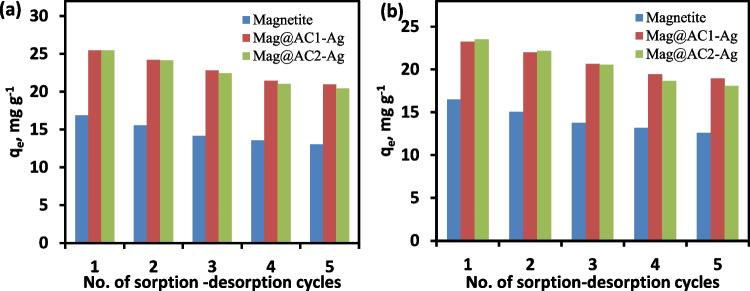
Reusability of magnetite, Mag@AC1-Ag, and Mag@AC2-Ag for the removal of Pb^+2^ (**a**) and Cd^+2^ (**b**)

The potential for sorbents’ regeneration has gained economic significance due to the high cost of wastewater treatment systems. For desorption process, 10 mL of 0.1 mol L^−1^ HCl was mixed with 0.05 g Pb- or Cd-loaded nanosorbent for 1 h at 25 °C. HCl is universally employed for desorbing many metal ions from the sorbents since it is frequently used in industry, metal ions are soluble in it, and it is relatively inexpensive (Jain et al. 2018). The desorbed Pb^+2^ and Cd^+2^ ions were evaluated, and the desorption efficiencies for Pb^+2^ using HCl were 93.33, 97.2, and 96.6%, while for Cd^+2^, they were 92.56, 95.7, and 94.4% for magnetite, Mag@AC1-Ag, and Mag@AC2-Ag, respectively. The sorption–desorption for Pb^+2^ and Cd^+2^ was performed repetitively, up to five cycles. As illustrated in Fig. 8, after each cycle, the sorption capacity was slightly reduced, indicating that the nanosorbents had good regeneration capability. For example, in the case of Mag@AC1-Ag, the sorption capacities of the first, second, and 3rd cycles for Pb^+2^ were 25.45, 24.20, and 22.80 mg g^−1^, respectively, and for Cd^+2^, they were 23.23, 22.00, and 20.65 mg g^−1^, respectively.

### Comparison with other sorbents

The characteristics of some adsorbents and their sorption capacities towards Pb^+2^ and Cd^+2^ are recorded in Table S1. The difference in the sorption capacities was a consequence of the distinctive characteristics of each adsorbent concerning the main functional groups, surface area, pore volume, etc. As could be seen, Mag@AC1-Ag and Mag@AC2-Ag exhibited good sorption capacities when compared to other sorbents.

### Antimicrobial activity of nanosorbent*s*

**Fig. 9:**
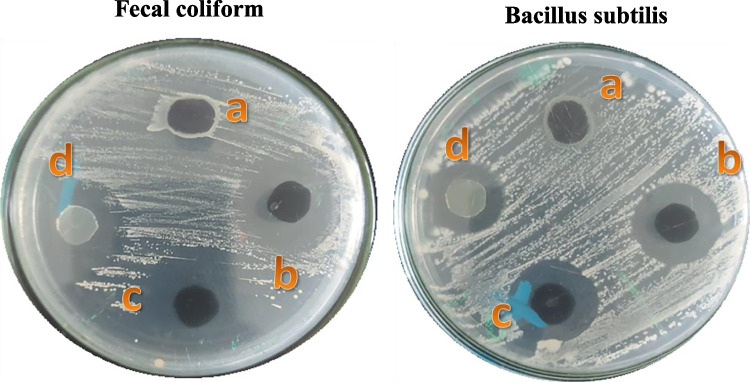
The inhibition zones of (a) magnetite, (b) Mag@AC2-Ag, (c) Mag@AC1-Ag, and (d) AgNO_3_ against fecal coliform and *Bacillus subtilis*

The antibacterial activity of magnetite, Mag@AC1-Ag, Mag@AC2-Ag and AgNO_3_ was examined by the zone of inhibition method against both fecal coliform (gram-negative) and *Bacillus subtilis* (gram-positive). Figure 9 and Table 5 display the results. As seen, magnetite had a little antibacterial activity against both strains. On the other hand, Mag@AC1-Ag, Mag@AC2-Ag, and AgNO_3_ exhibited high antimicrobial activity against both fecal coliform and *Bacillus subtilis*; however, they were more active against gram-negative than positive one. This could be as a result of the two strains’ cell walls having different compositions (Jemal et al. 2017). In contrast to gram-negative bacteria, which have cell walls made up of a thin peptidoglycan layer and a lipopolysaccharide layer, gram-positive bacteria have thick peptidoglycan layers made of linear polysaccharide chains cross-linked via short peptides, creating a three-dimensional rigid structure that makes nanosorbent penetration difficult. The lipopolysaccharides are comprised of negatively charged polysaccharides and covalently linked lipids, providing a poor permeability barrier to the positively charged nanosorbents due to the presence of AgNPs. Silver nanocomposites demonstrated significantly higher effluent disinfection efficiency and antibacterial activity against a wide range of microorganisms (Najafpoor et al. 2020). Upon contact with microbes, the silver nanocomposites are believed to be oxidized into Ag^+^ ions that destabilize the penetrability and respiration cellular functions and enter the microorganisms (Furlan et al. 2017), affecting intracellular processes such as DNA, RNA, and protein synthesis. This leads to cell death or cellular inactivation (Franci et al. 2015; Notriawan et al. 2013). Furthermore, other pathways have been identified, such as the interaction of silver nanocomposite with bacterial cell surface structures and the interaction of liberated Ag ions with sulfur and phosphorous in cell macromolecules (Ghaseminezhad et al. 2018). AgNPs can demonstrate their antibacterial action via the creation of reactive oxygen species, which can break down DNA and proteins in bacterial cells (Azócar et al. 2019).

**Table 5:** Inhibition zone diameters of Mag@AC1-Ag, Mag@AC2-Ag, and different materials against different bacterial strains

Material	Bacterial strains		Inhibition zone (mm)	Reference
	Gram negative	Gram positive		
Co_0.7_Zn_0.3_Fe_2_O_4_/PET/Ag	*Escherichia coli*	-	12.50	Jalali et al. (2017)
	-	*Staphylococcus aureus*	11.50	
AgNP-AC	*Escherichia coli*	*-*	18.00	Karthik and Radha (2016)
	-	*Bacillus subtilis*	17.00	
AgAC	*Escherichia coli*	-	10.60	Karadirek and Okkay (2019)
	-	*Staphylococcus aureus*	13.38	
GO–Ag NPs	*Escherichia coli*	-	20.00	Truong et al. (2022)
	-	*Staphylococcus aureus*	31.00	
AgNPAC	*Escherichia coli*	*-*	24.10	Altintig et al. (2022)
	*-*	*Staphylococcus aureus*	34.10	
AgNPs	*Pseudomonas aeruginosa*	*-*	24.40	Gopinathan and Balasubramanian (2022)
	*-*	*Bacillus cereus*	18.90	
Mag@AC1-Ag	Fecal coliform	-	28.00	This study
	-	*Bacillus subtilis*	25.00	
Mag@AC2-Ag	Fecal coliform	-	26.00	This study
	-	*Bacillus subtilis*	22.00	

Table 5 also displays the antibacterial activities of some other reported studies. It can be remarked that the values of the inhibition zones of Mag@AC1-Ag and Mag@AC2-Ag were smaller than those obtained by Truong et al. (2022) and Altintig et al. (2022) for gram-positive bacteria but higher for gram-negative bacteria. On the other hand, they were higher than the other materials for both strains, demonstrating the effectiveness of Mag@AC1-Ag and Mag@AC2-Ag as antibacterial materials.

### Application of real samples

To assess the analytical applicability of the produced nanosorbents for water remediation, magnetite, Mag@AC1-Ag, and Mag@AC2-Ag nanosorbents were applied to remove Pb^+2^ and Cd^+2^ ions from spiked water samples, including tap water, Nile water, and groundwater. The samples were spiked with Pb^+2^ and Cd^+2^ metal ions at a concentration of ≈ 50 mg L^−1^. The values of removal percentages (R%) and RSD% are given in Table 6. As shown, the higher values of the removal percentages of Pb^+2^ and Cd^+2^ from the tap, Nile, and groundwater samples indicated the applicability of the Mag@AC1-Ag and Mag@AC2-Ag nanosorbents as effective and eco-friendly sorbents to remove Pb^+2^ and Cd^+2^ ions from real samples. Tap water, as the clean matrix, resulted in the highest removal percentage compared to river water and groundwater, whose matrices were more complex than tap water (Nodeh et al. 2019).

**Table 6:** Removal of Pb^+2^ and Cd^+2^ from different water samples using magnetite, Mag@AC1-Ag, and Mag@AC2-Ag (*n* = 3)

Sample	Nanosorbent	Pb^+2^ (mg L^−1^)		R%	RSD%	Cd^+2^ (mg L^−1^)		R%	RSD%
		Before treatment	After treatment			Before treatment	After treatment		
Tap water	Magnetite	50.90	16.79	67.01	1.77	49.00	17.75	63.78	2.29
	Mag@AC1-Ag	50.90	0.29	99.44	3.22	49.00	3.13	93.61	2.84
	Mag@AC2-Ag	50.90	0.39	99.23	2.03	49.00	3.700	92.45	3.38
Nile water	Magnetite	50.90	17.23	66.15	1.60	49.00	18.55	62.14	1.35
	Mag@AC1-Ag	50.90	0.76	98.50	2.66	49.00	3.79	92.27	4.12
	Mag@AC2-Ag	50.90	0.91	98.20	2.47	49.00	4.37	91.08	1.67
Groundwater	Magnetite	50.90	17.61	65.40	1.65	49.00	19.13	60.96	1.88
	Mag@AC1-Ag	50.90	0.97	98.10	1.19	49.00	4.31	91.20	3.51
	Mag@AC2-Ag	50.90	1.19	97.67	0.84	49.00	4.90	90.00	1.67

## Conclusions

Using commonly available materials and equipment, eco-friendly mesoporous nanosorbents were produced via the incorporation of magnetite and antimicrobial silver nanoparticles into activated carbon-based palm shell. The obtained isotherms of the prepared samples, including magnetite, Mag@AC1-Ag, and Mag@AC2-Ag, proved the formation of mesoporous structures. Also, they have a pseudo-spherical shape with particle sizes in the range of 6–10, 4–10, and 8–14 nm for magnetite, Mag@AC1-Ag, and Mag@AC2-Ag, respectively. The prepared nanosorbents were characterized and employed for the Pb^+2^ and Cd^+2^ removal from water and wastewater. The sorption capacity of Pb^2+^ was greater than that of Cd^2+^ because Pb has a smaller hydrated ionic radius. The experimental data for Pb^+2^ and Cd^+2^ removal fitted well with the Langmuir isotherm. According to the kinetic studies, the pseudo-second-order model provided a superior fit to the obtained data. In addition, the thermodynamic parameters revealed that Pb^+2^ and Cd^+2^ removal using the nanosorbents was endothermic and spontaneous. The results further demonstrated the acceptable stability and significant recyclability of the nanosorbents. They exhibited higher efficiency for wastewater disinfection, where they showed strong antibacterial activity against fecal coliform and *Bacillus subtilis*. Furthermore, the prepared nanosorbents can be considered potential eco-friendly sorbents since they exhibited efficient elimination of toxic Pb^+2^ and Cd^+2^ ions from real samples, such as tap water, Nile water, and groundwater. In summary, the findings demonstrated that these eco-friendly nanosorbents would make better candidates for wastewater treatment.

## Supplementary Information

Below is the link to the electronic supplementary material.

Supplementary file1 (DOCX 163 KB)

## Data Availability

Raw data are available upon request.
