# Supplementary material for: Mesoporous Ag-functionalized magnetic activated carbon-based agro-waste for efficient removal of Pb(II), Cd(II), and microorganisms from wastewater
Source: Environ Sci Pollut Res Int. 2023 Mar 2;30(18):53548–65. doi: 10.1007/s11356-023-26000-w (PMC10119269; doi:10.1007/s11356-023-26000-w)
Supplement: Supplementary file 1 — Supplementary file1 (DOCX 163 KB) [file 11356_2023_26000_MOESM1_ESM.docx]

**Supplementary information**

**Mesoporous Ag-functionalized magnetite-activated carbon- based agro-waste for efficient removal of Pb(II), Cd(II), and microorganisms from wastewater**

Omnia I. Ali^1, *^, Eman R. Zaki^2^, Mohga S. Abdalla^1^, Saber M. Ahmed^2^

*^1^Chemistry Department, Faculty of Science, Helwan University, 11795, Cairo, Egypt*

*^2^Soil, Water and Environment Research Institute, Agriculture Research Centre, Giza, Egypt*

** Corresponding author: Tel.: 002 01002583552; fax: 00202 25552468*

*E-mail addresses:* [omniaali@science.helwan.edu.eg](mailto:omniaali@science.helwan.edu.eg) & [*omniaibrahim95@gmail.com*](mailto:omniaibrahim95@gmail.com)

ORCID/ 0000-0002-4205-0060


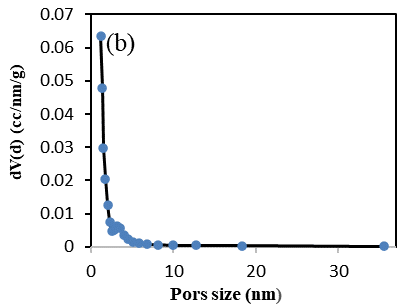

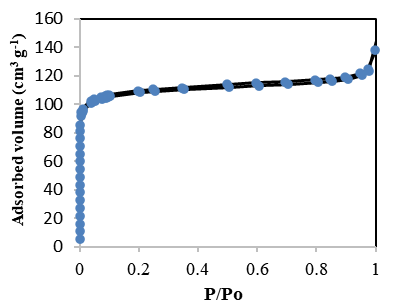

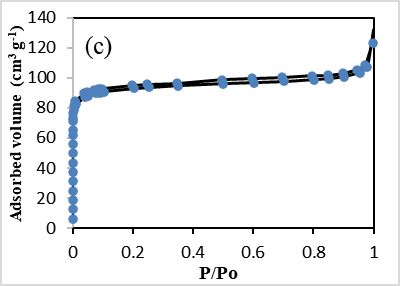

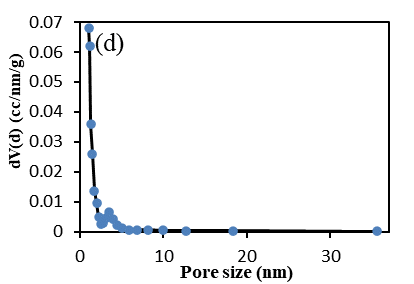


(a)

**Fig. S1** N_2_ adsorption–desorption isotherms and particle size distribution of AC1 (a,b) and AC2 (c,d), respectively.

**Fig. S2** Pseudo-first-order plots for the removal of Pb^+2^ using magnetite, Mag@AC1-Ag, and Mag@AC2-Ag.

**Fig. S3** Pseudo-first-order plots for the removal of Cd^+2^ using magnetite, Mag@AC1-Ag, and Mag@AC2-Ag.

**Fig. S4** Pseudo-second-order plots for the removal of Pb^+2^ using magnetite, Mag@AC1-Ag, and Mag@AC2-Ag.

**Fig. S5** Pseudo-second-order plots for the removal of Cd^+2^ using magnetite, Mag@AC1-Ag, and Mag@AC2-Ag.

**Fig. S6** Boyd plots for the removal of Pb^+2^ using magnetite, Mag@AC1-Ag, and Mag@AC2-Ag.

**Fig. S7** Boyd plots for the removal of Cd^+2^ using magnetite, Mag@AC1-Ag, and Mag@AC2-Ag.

**Fig. S8** Langmuir isotherm plots for the removal of Pb^+2^ using magnetite, Mag@AC1-Ag, and Mag@AC2-Ag.

**Fig. S9** Langmuir isotherm plots for the removal of Cd^+2^ using magnetite, Mag@AC1-Ag, and Mag@AC2-Ag.

**Fig. S10** Freundlich isotherm plots for the removal of Pb^+2^ using magnetite, Mag@AC1-Ag, and Mag@AC2-Ag

**Fig. S11** Freundlich isotherm plots for the removal of Cd^+2^ using magnetite, Mag@AC1-Ag, and Mag@AC2-Ag.

**Fig. S12** Dubinin-Radushkevich isotherm plots for the removal of Pb^+2^ using magnetite, Mag@AC1-Ag, and Mag@AC2-Ag.

**Fig. S13** Dubinin-Radushkevich isotherm plots for the removal of Cd^+2^ using magnetite, Mag@AC1-Ag, and Mag@AC2-Ag.

**Fig. S14** Temkin isotherm plots for the removal of Pb^+2^ using magnetite, Mag@AC1-Ag, and Mag@AC2-Ag.

39

**Fig. S15** Temkin isotherm plots for the removal of Cd^+2^ using magnetite, Mag@AC1-Ag, and Mag@AC2-Ag.

**Fig. 16** Thermodynamic plots for the removal of Pb^+2^ using magnetite, Mag@AC1-Ag, and Mag@AC2-Ag.

**Fig. 17** Thermodynamic plots for the removal of Cd^+2^ using magnetite, Mag@AC1-Ag, and Mag@AC2-Ag.

Table S1 the characteristics of some adsorbents and their sorption capacities towards Pb^+2^ and Cd^+2^.

| Adsorbent | Adsorbent characteristics | | | | Sorption characteristics | | | Reference |
| --- | --- | --- | --- | --- | --- | --- | --- | --- |
|  | Surface area (m^2^ g^-1^) | Pore volume (cm^3^ g^-1^) | Type of adsorbent | Type of isotherm | Kinetics | q_max_ (mg g^-1^) | |  |
|  |  |  |  |  |  | Pb^+2^ | Cd^+2^ |  |
| Magnetite/ powder activated carbon | 671.20 | 4.87 | Mesoporous | IV | Pseudo second-order | 71.42 | - | (Kakavandi et al., 2015) |
| Magnetic activated carbon/clay composite | 174.57 | 0.31 | - | - | Pseudo  second-order | 143.56 | - | (Le et al., 2019) |
| Fe_3_O_4_@sawdust carbon | 14 | 0.086 | - | - | Pseudo  second-order | 151.5 | - | (Kataria et al., 2022) |
| Chitosan- Fe_3_O_4_-Modified Fish Bone Char | 19.287 | 0.047 | Mesoporous | IV | Pseudo  second-order | - | 64.31 | (Yang et al., 2022) |
| Magnetic iron oxide/ sawdust carbon | 30.15 | 0.152 | - | - | Pseudo  second-order | - | 51 | (Kataria & Garg, 2018) |
| Activated carbon of lemon/Fe_3_O_4_ | 38.7 | - | Mesoporous | IV | Pseudo  second-order | - | 39.6 | (Peighambardoust et al., 2021) |
| Oak wood ash/GO/ Fe_3_O_4_ | 85.8 | 0.395 | Mesoporous | IV | Pseudo  second-order | 47.16 | 43.66 | (Pelalak et al., 2021) |
| Magnetic (MnFe_2_O_4_) activated carbon | 553.3 | 0.374 | Mesoporous | IV | Pseudo  second-order | 253.2 | 73.3 | (Zhang et al., 2021) |
| Magnetic chrysotile nanotubes | 162.0 | 0.32 | - | IV | Pseudo  second-order | 23.67 | 23.82 | (Yu et al., 2015) |
| Mag@AC1-Ag | 220.9 | 0.31 | Mesoporous | IV | Pseudo  second-order | 75 | 62.5 | This study |
| Mag@AC2-Ag | 203.6 | 0.28 | Mesoporous | IV | Pseudo  second-order | 70 | 60 | This study |
